# Workforce scheduling efficiency assessment in construction projects through a multi-objective optimization model in the COVID-19 context

**DOI:** 10.1016/j.heliyon.2023.e16745

**Published:** 2023-06-03

**Authors:** Federico Corral, Eric Forcael, Rodrigo Linfati

**Affiliations:** a Department of Industrial Engineering, Universidad del Bío-Bío, Concepción, Chile; b Department of Civil and Environmental Engineering, Universidad del Bío-Bío, Concepción, Chile

**Keywords:** Workforce, Scheduling, Construction projects, Multi-objective optimization, COVID-19

## Abstract

The COVID-19 disease has caused a drastic stoppage in the construction industry as a result of quarantines. For this reason, this study focuses on the workforce scheduling problem when working under COVID labor distancing constraints, and additional costs derived from deviation hours or hiring new employees that managers must assume on a project due to circumstances. A multi-objective mixed integer linear programming model was developed and solved using weighting and epsilon constraint methods to evaluate workforce scheduling and the mentioned COVID costs. The first objective function corresponds to the sum of the total extra hours; the second objective function represents the total non-worked but paid hours. Two sets of experiments are presented, the first based on a design of experiments that seeks to determine the relationship between the proposed objective functions and a methodology to determine the cost of considering COVID constraints. The second set of experiments was applied in a real company, where the situation without COVID vs with COVID, and without allowing extra hours vs with COVID allowing extra hours were compared. Obtained results showed that hiring additional employees to the man-crew leads the company to increase the extra hours cost up to 104.25%, being more convenient to keep a workforce baseline and to pay extra hours costs. Therefore, the mathematical model could represent a potential tool for decision-making in the construction sector, regarding the effects of COVID-19 costs on workforce scheduling construction projects. Consequently, this work contributes to the construction industry by quantifying the impact of COVID-19 constraints and the associated costs, offering a proactive approach to address the challenges posed by the COVID-19 pandemic for the construction sector.

## 1 Introduction

In December 2019, the COVID-19 disease was discovered and since then it has spread around the world [1]. Several types of research have indicated that a fundamental aspect of early COVID-19 outbreak control is travel restrictions [2,3]. Others claim that staying at home is one of the most effective ways to keep the disease under control [4,5]. For this reason, quarantines, and entry barriers, among others, have been implemented [6] to increase social distancing to reduce the infection risk mobility to the minimum possible [7]. Despite these efforts, the pandemic has caused not just a serious global health crisis, but also both economic and industrial ones [8]. In particular, the construction industry has been severely interrupted [7], due to construction worker shortages and stoppages in construction projects [9]. For this reason, the following study hypothesizes that it is possible to determine an efficiency relationship between deviation hours and total employee numbers for a construction project under COVID constraints, to find the best solution for decision-makers between paying extra hours costs or hiring additional employees.

### 1.1 COVID-19 on the construction industry

According to the International Labor Organization (ILO), before the pandemic the construction sector represented around 7.7% of world employment [10], projecting 13.4% of the World GDP [11]. However, in the current COVID-19 context, the prognosis for the growth of this sector has been recently reviewed downward to 0.7% with the possibility of further cuts [12]. Falling revenues and growing challenges in project development have hindered many sectors such as construction, with a corresponding negative impact on the workforce. As an example, approximately 7.5 million construction workers were employed in the United States in 2018. However, the unemployment rate for this sector was at a rate of 16.6% according to the US Bureau of Labor Statistics [13]. Similar examples to the United States are found throughout the world, which emphasize the importance of the construction industry in the economic growth of countries [14]. On the other hand, diverse efforts have been made to generate models to sustainable allocate the construction workforce for work resumption during COVID-19 [15] or to apply multi-objective optimization approaches in areas such as transport engineering to recover from COVID-19 disruption [16]. Other examples are found in terms of understanding the impact of COVID-19 on construction projects in developing countries [17] or how to manage the construction workforce based on lean construction in the context of COVID-19 [18]. However, there is limited evidence that aspects such as the relationship between workforce scheduling with potential extra costs, derived from pandemic contexts, have been assessed in contrast to normal operating conditions. However, aspects such as the relationship between workforce scheduling with potential extra costs, derived from pandemic contexts, have not been assessed in contrast to normal operating conditions.

In general, current operational restrictions along with a contagion fear in construction reached a point where many companies —especially small and medium enterprises—, were facing serious liquidity and sustainability problems, and even risking bankruptcy in developing countries [19]. Because of the latter, several mechanisms have been developed to better manage the impact of their project and employees, by evaluating contractual measures that exempt from liability for non-compliance due to force majeure [20,21]. Today, despite the COVID-19 outbreak has had a significant influence on the construction industry, construction firms have reopened and operated in the face of a virus that has remained a big threat to the industry and economies [22].

### 1.2 Safety measures to protect the workforce

First of all, it has to be emphasized the importance of safety performance in the construction industry [23]. Within a pandemic context, depending on the severity of the crisis, the contingency measures that have affected the construction sector have varied according to location and type of project. In some countries such as China and Italy, certain construction activities were considered essential, for example, those related to hospital installation [24]. On the other hand, countries such as Austria, Barbados, and Russia opted for the partial or total stoppage of projects, whose work closures led to prioritizing reopening after the first contagion wave [25]. In countries like Panama, the reopening of projects with up to 500 employees was determined, subject to working hour restrictions [25]. Particularly, the design of this research was focused on a country with strict and extended quarantines.

Another important issue refers to the pandemic impact on classification levels in this particular sector. In general terms, these can be classified as off-site impacts, e.g. contractual consequences of the COVID-19 context; and on-site impacts, e.g. on-site employees' safety and health [26,27]. Occupational health and safety risk control measures are essential to reduce risks generated by the construction project's activities [28]. However, there is a greater viral exposure risk for those who must work on-site [29]. In detail, on-site employees risk their health while moving, mainly due to transport overcrowding and the absence of risk control measures.

Given the impact of COVID-19 on all actors comprised in the construction sector, prevention, and control measures have played a relevant role in promoting economic reactivation in a safe way [30]. Risk control has considered the adoption of daily measures, including social distancing, mask use, hand washing, temperature control for visitors and workers, and workers training [9,13]. In addition, actions have been taken to reduce and eliminate, if possible, any close contact between workers of the same sector, by implementing rotating shifts, contact tracing, and containment procedures for those employees with suspected COVID symptoms, among others [10]. Even when the construction industry has adjusted its operations given the impossibility of replacing on-site activities with remote alternatives [9], recent studies have quantified the number of infected employees during project execution at 30–90% [29]. Therefore, optimizing the workforce scheduling, on healthy and available employees, corresponds to a critical step for any construction project regarding compliance with contract deadlines and cost overruns.

### 1.3 Problems related to workforce scheduling

In general, the completion of a construction project according to a given time is one of the major objectives for project managers [31], where delays are commonly normalized in the construction sector [32]. To deal with these facts, several multi-objective optimization methods have been used in a wide range of construction-related research [33,34,35,36,37,38,39]. In regards to prefabricated composite construction, Sojobi & Liew [40] used a Taguchi-RSM (Response Surface Methodology) multi-objective approach to compare the cost-effectiveness of optimum bio-inspired CFRP (Carbon Fiber Reinforced Polymer) composite reinforcement configurations. For repetitive scheduling, a multi-objective model was created by Salama & Moselhi [41] that utilized CCPM (Critical Chain Project Management) to address the uncertainties associated with production rates, work quantities, and resource availability. Ozcan-Deniz & Zhu [33] studied the greenhouse gas emissions in highway construction projects through multi-objective optimization. Anvari et al. [42] employed a multi-objective model in manufacturing industries to deal with duration alternatives, cost and time, the energy consumption of idle time, and turning on/off of machinery, enabling production managers to choose an appropriate solution from the optimized set derived from Pareto optimal solutions.

Siu et al. [43] proposed a schedule assessment approach based on characterizing budget sufficiency metrics and resource utilization metrics to assess multiple alternatives of resource-constrained project schedules objectively. Other researchers established that, for obtaining a successful project scheduling, resource allocation and time-cost trade-offs must be studied regarding each other [44]. Goli et al. [45] addressed a multi-objective closed-loop supply chain network design with uncertain paraments, to maximize the total created jobs in the supply chain. Others designed a multi-objective mixed integer linear programming model to design a sustainable closed-loop supply chain network of face masks during the COVID [46]. Power et al. [47] developed a study on healthcare workers by mapping the studies about factors associated with sleep characteristics in this type of employees working during the pandemic and then examining the availability of information on the influence of atypical work schedules. Lehnig *et at.* [48] quantified the impact of alternative break schedules on cumulative COVID-19 incidence on university campuses, by using student mobility data and Monte Carlo simulations of returning infectious student size. Finally, Guerriero & Guido [49] proposed optimization models to address flexible staff scheduling problems during the COVID pandemic, taking into account demand requirements, employees' personal and family responsibilities, and anti-COVID measures.

The impact of various resource utilization plans has been assessed on project performance to seek an optimal/near-optimal solution by optimizing quality, cost, and time [50]. Senouci & Adeli [51] presented a mathematical model for resource scheduling by considering project scheduling characteristics such as precedence relationships, multiple crew strategies, and time cost trade-off, where resource leveling and resource-constrained scheduling are performed at the same time.

Karim & Adeli [52] elaborated an object-oriented (OO) information model for construction scheduling, cost optimization, and change order management, which could provide support for schedule generation and review, cost estimation, and cost-time trade-off analysis. Later, the same authors presented the implementation of the information model, in a prototype software system called CONSCOM for the scheduling and management of construction projects with cost-optimization capability, which is implemented in Visual C++. In the book *Construction Scheduling, Cost Optimization, and Management: A New Model Based on Neurocomputing and Object Technologies,* Angelou [53] summarized an approach to construction scheduling, cost optimization, and construction management by using neural networks and object-oriented software.

This research focuses on the workforce scheduling problem for construction managers and decision-makers caused by the lack of workers due to sanitary and travel restriction barriers imposed as public policies during the pandemic scenario. From this point, what decision to take between paying for deviation hours costs or hiring additional employees, considering EPPs for both scenarios? In summary, being mandatory costs that must be assumed to execute construction projects under COVID constraints. The latter is especially relevant due to the adoption of Dynamic quarantines. In detail, a public health response established in Chile to mitigate the spread of the COVID disease by restricting the activities and movement of people, organizations, and companies [54]. The objective is to generate social distancing, being prolonged or lifted based on weekly evaluations and depending on the relative number of infected individuals in each city or commune, also known as administrative subdivisions [55].

In response to this problem description, this article proposes a multi-objective optimization mathematical model capable of assessing a construction project's efficiency, considering the employee's workforce scheduling and the mentioned COVID costs, during the on-site work execution in a pandemic context. Results were obtained after applying a multi-objective integer programming model in an experiment set, and then in a construction project performed by a Chilean construction company. Thus, the main contribution of this research is to emphasize the idea that quantifying the influence of COVID constraints with potential costs may help construction managers on the workforce scheduling to run a construction project according to its Gantt Chart, project budget, and deadlines. It is important to indicate that the mathematical model provides a preventive solution rather than a reactive one, by avoiding COVID positive cases or closed contacts among workers of the construction project. Furthermore, there are no publications that study the relationship between extra and non-worked by paid hours on workers from the construction sector, nor a comparison between paying deviation hours costs in a man-crew baseline, versus hiring additional employees costs directly associated with the COVID event. The proposed methodology was designed for a country with massive and extended quarantines. Specifically, focused on places where people were prohibited from going out of their homes, just being able to do it in a limited way, to meet basic needs and perform critical work. In detail, the methodology considers the data collected from a construction project such as the number of weeks, number of shifts, number of working areas, worker's availability, and max/min working hours per week. Based on this data, the mathematical model optimizes workforce scheduling. Finally, the application of the scheduling, according to the non-dominated solutions obtained from a Pareto frontier, results in the minimization of the COVID cost related to deviation hours, which are extra and non-worked but paid hours.

The remainder is organized as follows: In Section 2 scheduling and multi-objective models are described, while in Section 3 the developed mathematical model is presented. In Section 4, experiments are extensively described from a theoretical and practical approach to study the relationship between the proposed objective functions and estimate COVID costs. Finally, in Section 5 concluding remarks are discussed.

## 2 Materials and methods

The proposed methodology integrates scheduling and multi-objective optimization approaches with a novel mathematical model to contribute to the field of construction management by addressing workforce scheduling efficiency in the context of the COVID-19 pandemic. The methodology offers a proactive approach to tackling the challenges posed by the pandemic and provides valuable insights for decision-making in the construction industry. It consists of the following steps:

Problem Formulation: The first step involves mathematically formulating the scheduling problem, which is a crucial process in business decision-making [51,52]. The problem is defined, taking into account the specific constraints and objectives of the construction project [57], including the COVID-19-related constraints and associated costs. This step establishes the foundation for the subsequent optimization techniques.

Multi-Objective Optimization: The methodology employs a multi-objective optimization approach, which has been applied to a variety of construction projects [50,57,58,59] [47,48,49,50], to find a set of non-dominated solutions that form the Pareto frontier [61,62]. Then, a set of solutions belonging to the feasible region are determined [63]. Two commonly used methods [64], the Weighting and the Epsilon constraint methods (ε – constraint), are employed in this study. The Weighting method creates a single-objective problem based on the multi-objective problem and assigns weights to each objective function based on the decision-maker's preferences. The Epsilon constraint method involves incorporating constraints on the objective functions to determine a subset of Pareto optimal solutions.

Design of Experiments: A design of experiments was conducted to determine the relationship between the proposed objective functions and the cost of considering COVID-19 constraints. The DOE systematically varies the weights and constraints in the mathematical model to analyze their effects on the resulting solutions and identify the trade-offs between different objectives and costs. This step helps in understanding the sensitivity of the solutions to different factors.

Real-World Case Study: The methodology is applied to a real-world case study in a construction company to compare scenarios with and without COVID-19 constraints and extra hours. Actual data from the construction project, including workforce scheduling data, COVID-19-related constraints, and associated costs, are used in the case study to validate the effectiveness of the proposed methodology in a practical setting. This step ensures the practical applicability of the methodology.

Analysis and Evaluation: The solutions obtained from the multi-objective optimization are analyzed using decision-making tools to identify the most preferred solutions based on decision-makers preferences and the project's specific requirements. Finally, the optimized workforce scheduling plan is evaluated in the construction project in terms of meeting the project objectives, adhering to COVID-19-related constraints, and minimizing the associated costs.

In summary, the methodology used in this study involves problem formulation, multi-objective optimization, design of experiments, real-world case study analysis, and evaluation of the optimized scheduling plan. Furthermore, the novel mathematical model proposed is an innovative tool due to its industrial application. Therefore, this comprehensive approach allows for effectively addressing the workforce scheduling problem in the context of the COVID-19 pandemic and provides valuable insights for decision-makers in the construction industry.

## 3 Model formulation

**Fig. 1:**
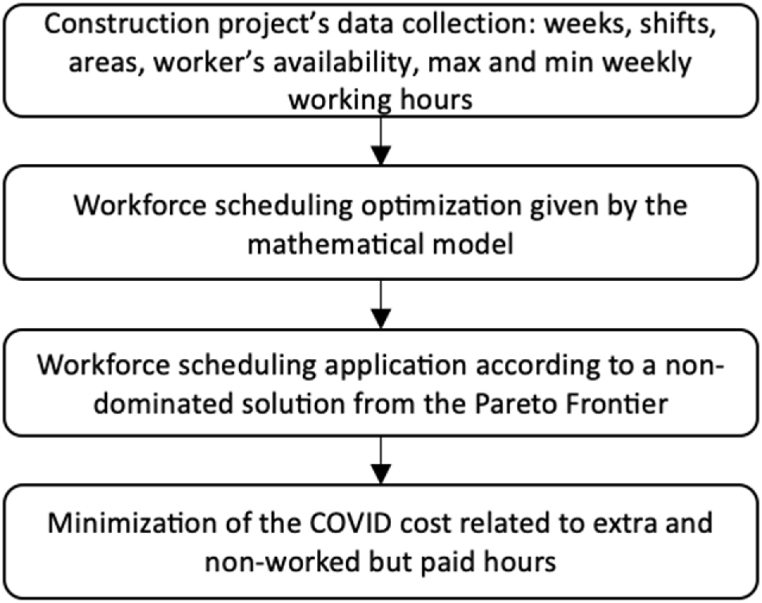
Scheduling workforce optimization for a construction project under the COVID context.

To pose a scheduling problem applied to a construction project during the COVID-19 contingency, a Mixed Integer Programming model (MIP) will be used. For practical purposes, the procedure is depicted in Fig. 1.

The proposed mathematical model seeks to reduce deviation hours that lie in the workforce scheduling. That means, the total extra hours associated with productive work, and the total non-worked but paid hours associated with unproductive work. The model considers a contractual hours number, a maximum extra hours number, and a demand for working hours according to the construction project design. Given the COVID-19 situation, it is fundamental to reduce the possibility of close contact to prevent a massive number of sick workers. With this in mind, it is sought that employees can be only in one area per shift, and be in the same area for two weeks, but in different shifts to restrict worker's mobility. For the proposed model, the following sets were defined:

**Table:** 

I	Let i∈I be a set of weeks of the project
J	Let j∈J be a set of work areas
K	Let k∈K be a disjoint set of shifts, $K=K_{1}⋃K_{2}$.
$K_{1}$	Let $k\in K_{1}$ be a set of regular shifts, $K_{1}\subset K$.
$K_{2}$	Let $k\in K_{2}$ be a set of Saturday shifts, $K_{2}\subset K$.
L	Let l∈L be a set of workers

In this problem, it was considered that regular and Saturday shifts have a maximum number of weekly working hours per area, which is represented here by the parameter $\lambda_{kj}$. To establish a balance of activities and guarantee a productivity level for each area, it was defined parameter $\tau_{kj}$ as the minimum number of weekly working hours per shift and area imposed, according to the construction project design. Finally, the binary parameter $c_{lj}$ was set to indicate if a worker can work in a certain area depending on his/her specialty. In detail, the parameters were defined as follows:

**Table:** 

$\lambda_{kj}$	Maximum number of weekly working hours for each work shift k and work area j
$\tau_{kj}$	Minimum number of weekly working hours for each work shift k and work area j
$H_{l}^{\max}$	Maximum weekly contractual working hours per worker l
$c_{lj}$	{1, if worker l can work in area j, according to specialty 0, otherwise
Pw	Total number of project weeks
BigM	Large number
Finally, the following decision variables were defined:	
$x_{kl}^{ij}$	{1, if worker l is assigned to the shift k to work in area j in week i 0, otherwise
$y_{kl}^{ij}$	Number of working hours spent in area j by worker l assigned to shift k in the week i
$F_{li}^{+}$	Number of extra hours associated with worker l in the week i
$F_{li}^{-}$	Number of non-worked hours associated with worker *l* in the week i

### 3.1 Mathematical model formulation

The objective function of the proposed model seeks to minimize the sum of extra hours ${(F}_{li}^{+})$, as shown in Equation (1), and non-worked hours that must be paid ${(F}_{li}^{-})$, Equation (2). In detail, variable $F_{li}^{+}$ is defined as the total number of extra hours performed by a worker for a certain week of the construction project. This variable is associated with the concept of productive work, which represents the amount of time where value-adding activities are done [65]. On the contrary, variable $F_{li}^{-}$ corresponds to the total number of non-worked hours that must be paid, per worker and week. Then, it is related to unproductive work as the amount of non-value adding activities during the construction on-site process which gives rise to delays in the project's Gantt Chart [65]. It is important to notice that these two objective functions are in conflict because, the more contributory activities are performed, the less waste of time is obtained from workers for the project, and vice versa [65]. In practical terms, equations (1), (2) minimizes the total deviation hours that lie in the workforce scheduling, in terms of the maximum contractual weekly working hours performed by worker given the COVID-19 situation, which is represented here as a parameter $H_{l}^{\max}$, and according to the number of working hours spent in an area by a worker assigned to a regular or saturday shift, which is represented here as parameter $y_{kl}^{ij}$. Therefore, the objective function leads to reduce both productive and unproductive work costs. This is defined as follows:

(1) $$\mathrm{Min}\;z_{1}=\sum_{l\in L}\sum_{i\in I}F_{li}^{+}$$

(2) $$\mathrm{Min}\;z_{2}=\sum_{l\in L}\sum_{i\in I}F_{li}^{-}$$

The constraints associated with the model consider aspects of workforce scheduling on-site, minimum and maximum working hours, and COVID-19 measures to minimize the contagion risk. These three constraint families are disaggregated below in Equation (3) and (4), (5), (6), (7), (8), and (9), (10), (11), (12).

(3) $$\sum_{j\in J}\sum_{k\in K_{1}}x_{kl}^{ij}\leq 1\;i\in I,l\in L$$

(4) $$x_{kl}^{ij}=0\;i\in I,j\in J,k\in K,l\in L:C\left[l,j\right]=0$$

The constraint shown in Equation (3) indicates that each worker can be weekly assigned to a maximum of one shift k and one area j. On the other hand, the constraint shown in Equation (4) ensures the compatibility of assigning worker l to perform functions in area j, according to specialties.

(5) $$y_{kl}^{ij}\leq BigM*x_{kl}^{ij}\;i\in I,j\in J,k\in K,l\in L$$

(6) $$y_{kl}^{ij}\leq \left(\lambda_{kj}*x_{kl}^{ij}\right)+F_{li}^{+}\;i\in I,j\in J,k\in K,l\in L$$

(7) $$\sum_{l\in L}y_{kl}^{ij}\geq \tau_{kj}\;i\in I,j\in J,k\in K$$

(8) $$\sum_{j\in J}\sum_{k\in K}y_{kl}^{ij}+F_{li}^{-}-F_{li}^{+}=H_{l}^{\max}\in I,l\in L$$

Equation (5) considers a BigM constraint to relate the integer variable $y_{kl}^{ij}$ with the binary $x_{kl}^{ij}$, so $x_{kl}^{ij}$ takes the value 1 when $y_{kl}^{ij}$ is greater than 0. The constraint that appears in Equation (6) sets a maximum amount of weekly working hours per worker, including extra hours. The constraint shown in Equation (7) guarantees that a minimum of total working hours per week is respected for each shift and area, for keeping the productivity level that requires the construction project. Finally, the constraint shown in Equation (8) marks the contractual amount of weekly working hours for each worker.

(9) $$\sum_{k\in K1}x_{kl}^{hj}=\sum_{k\in K1}x_{kl}^{\left(h+1\right)j}\;j\in J,l\in L,h\in I:h<Pw\;and\;h\;is\;odd$$

(10) $$\sum_{j\in J}\left(x_{kl}^{hj}+x_{kl}^{\left(h+1\right)j}\right)\leq 1\;l\in L,k\in \left\{1,2\right\},h\in I:h<hh\;and\;h\;is\;odd$$

(11) $$x_{ml}^{ij}\leq x_{nl}^{ij}\;l\in L,j\in J,i\in I,m\in K_{2},n\in K_{1}$$

(12) $$x_{ml}^{ij}=0\;i\in I,j\in \left\{2,3,4\right\},l\in L,m\in K_{2}$$

Moreover, the constraint shown in Equation (9) states that if worker l is assigned to area j in week h, they must work in the same area during the following week (h+1). The constraint expressed in Equation (10) requires each worker to work in a maximum of one area per shift. That is, if a worker is assigned to area 1 for the morning shift (k=1), he/she will not be able to work in the remaining areas. Thus, the constraint presented in Equation (11) establishes that a worker can work the Saturday shift ($K_{2}$) as long as he/she has been assigned to the morning shift and only for area 1. The constraint shown in Equation (12) forbids all workers to perform job activities in areas 2, 3, and 4 during the Saturday shift. Finally, the domain of the decision variables is defined in Equations (13), (14), (15).

(13) $$x_{kl}^{ij}\in \left\{0,1\right\}\;i\in I,j\in J,k\in K,l\in L$$

(14) $$y_{kl}^{ij}\geq 0\;i\in I,j\in J,k\in K,l\in L$$

(15) $$F_{li}^{+},F_{li}^{-}\geq 0\;l\in L,i\in I$$

### 3.2 Solution approach

The multi-objective function of the scheduling problem proposed to schedule the employee working hours who performs on-site activities, considered two functions whose sum seeks to be minimized. On the one hand, the sum of non-worked hours, and on the other hand, the sum of extra hours, according to employee per week in both cases. Based on the above, it was proposed to work the model through Weighting and Epsilon constraint (ε−constraint) methods. As mentioned in the previous section, the weighting method addresses MOP problems as a single objective to minimize the weighted sum of them. Therefore, the described function in Equation (3) is rewritten and shown in Equation (16):

(16) $$\mathrm{Min}\;\alpha *\sum_{l\in L}\sum_{i\in I}F_{li}^{+}+\beta *\sum_{l\in L}\sum_{i\in I}F_{li}^{-}$$

On the other hand, to use the ε−constraint method it is necessary to rethink one of the objective functions as a new constraint, being $F_{li}^{+}$ in this case, which is incorporated into the previously defined ones [64]. According to Tzortzopoulos et al. (2020) in the construction sector, non-worked hours paid are a more important variable than on-site extra hours. Finally, based on Equations (1), (2) the new objective function is rewritten in Equations (17), (18) as follows.

(17) $$\mathrm{Min}\;z=\sum_{l\in L}\sum_{i\in I}F_{li}^{-}$$

subjectto.

(18) $$\sum_{l\in L}\sum_{i\in I}F_{li}^{+}\leq \varepsilon$$

Equations 3–15

### 3.3 Experiments design

A series of four experiments were conducted to evaluate the proposed model consistency, as a tool for measuring COVID-19 effects on the construction sector. Each test was designed to analyze its efficiency, in terms of the ability to determine eventual COVID cost overruns, weigh the relative importance of extra and non-worked hours in the project, and demonstrate the new constraints’ relevance leading to minimizing contagions and avoiding on-site project stoppage risks. These experiments are described as follows.

#### 3.3.1 Experiment Nº1

To evaluate whether a construction project will work in terms of the prioritization of extra hours or non-worked but paid hours, a first experiment of the comparison of two work scenarios was designed. On the one hand, where the extra hours worked ($F_{li}^{+}$) are decisive for the project, and on the other, where more importance is given to non-productive hours ($F_{li}^{-})$. In methodological terms, *alpha* and *beta* weights were assigned to variables $F_{li}^{+}$ and $F_{li}^{-}$, respectively. From the obtained results, it was possible to highlight the importance of paying productive or non-productive hours, since in practice it translates into costs for hourly dedication, or poorly allocated monetary resources.

#### 3.3.2 Experiment Nº2

To evaluate the relevance of suitable work planning in terms of employee crews and labor hours during the COVID-19 situation, a second experiment was proposed consisting of assessing the effects of over and under estimations of the extra hours paid, in contrast to the required for the construction project design. Methodologically, the same data considered for experiment Nº1 was used, but applying a *gamma* multiplicative factor on the parameter $\tau_{kj}$. From the results, changes experienced by the extra hour curve were observed as a result of incorrect production planning required for workers, and the effects caused by COVID labor constraints. Likewise, how this issue generated an impact on the project costs in comparison with the original-basis condition, that is correct planning. Finally, conclusions regarding the behavior of non-worked but paid hours were developed.

#### 3.3.3 Experiment Nº3

To evaluate the efficiency relationship between paid hours according to the on-site work schedule of the construction project under the COVID-19 context, the ε−constraint method was implemented to obtain a frontier of efficient solutions which are exchanged between worked and non-worked hours. In practical terms, by running this third experiment a Pareto Frontier was originated on which it was possible to understand the effect of increasing extra hours by restricting idle hours during on-site work, and vice versa. On the other hand, it allowed discussion of the model viability for the construction sector, in terms of reducing labor imbalances, nowadays frequently given the need to safeguard the execution of this type of project, and thus avoid cost overruns.

#### 3.3.4 Experiment Nº4

To identify the possible arrival of COVID costs derived from new conditions imposed on the construction project, a numerical analysis related to COVID and non-COVID conditions was proposed as a fourth experiment. Methodologically, labor constraints considered in the model were eliminated to show potential COVID cost overruns during the project execution. The numerical analysis considered possible hourly variations derived from the relaxing of the COVID constraints, greater freedom of movement among areas, unrestricted work assignment between weeks, and general flexibility in human resource programming. From the results obtained, the practical significance that it represents for a construction company the emergence of additional costs for keeping the work in progress was analyzed. Finally, it allowed contrasting differences between extra and non-worked but paid hours for both assumed scenarios of the construction project.

### 3.4 Model application to the construction industry

A series of two practical experiments were conducted to apply the proposed model to a real construction project performed during the pandemic events. Due to the nature of the case study, local labor codes were considered, implying an adaptation of the model constraints. These experiments are described as follows.

#### 3.4.1 Experiment I

To evaluate the extra hours’ behavior when adding several employees per specialty to the original crew and vice versa, a first experiment was designed. Under the COVID constraint, the original 104-man crew was compared to two scenarios. In Scenario 1, employees cannot work extra hours on the 40-week construction project. In Scenario 2 the company seeks a balance between the number of workers and extra hours.

#### 3.4.2 Experiment II

To quantify the total COVID cost derived from applying the set of constraints (9)–(12), a second experiment was designed. In methodological terms, a total cost comparison was performed to identify possible cost increases derived from deviation hours, incomes, and sanitization/hygiene factors. This contrast considered the original baseline, a scenario without allowing employees to work extra hours, and another scenario that allows up to 10 weekly extra hours. For such purposes real data was used, corresponding to the worker's monthly incomes, and the COVID personal protective equipment (PPE) cost for a real construction project.

## 4 Results and discussion

Before the data analysis and discussion, it has to be noted that the mathematical model described above was implemented through the AMPL software, version 20210906. Although the software hosts a wide range of solvers, the mixed integer linear programming model was developed and solved through CPLEX 20.1.0. Results from the experiment design and the model application to the construction industry will be presented. For each set of the experiment, there will be a discussion.

### 4.1 Experiments design approach results

The data used for the proposed model considered a 30-week construction project with three shifts and four different working areas for a 40-man crew. In terms of effective hours per employee according to the crew, maximums of 30 and 40 weekly hours were considered under a proportion of 75.0% and 25.0%. They obey the idea of implementing a crew flexible schedule policy, given the difficulties of traveling to and from work. The description of shifts during the COVID-19 condition can be explained as follows. Workers may work from Monday to Friday, in the morning from 08 a.m. to 02 p.m. (shift 1), or in the afternoon from 02 p.m. to 10 p.m. (shift 2). Moreover, workers may work on Saturday but only during the morning from 08 a.m. to 02 p.m. (shift 3).

#### 4.1.1 Results from experiment Nº1

**Fig. 2:**
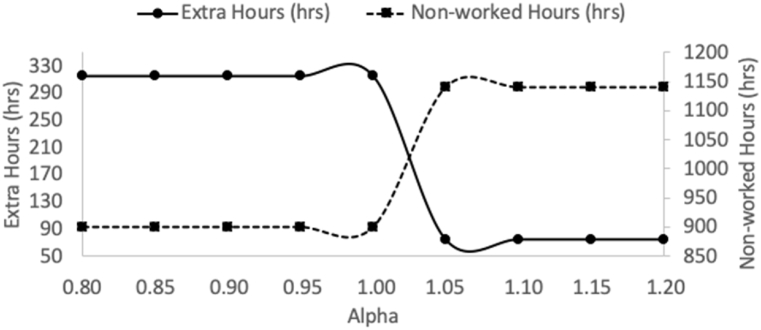
Chart with the analysis of the alpha weight (α) associated with the decision variable $F_{li}^{+}$, by using the Weighting Method.

The Weighting Method was used to evaluate the convenience of prioritizing the reduction of extra hours in contrast to non-worked hours but equally paid. Proposing a first scenario where the decrease in extra hours is transcendent for the on-site work, obtained results indicated that when increasing the weight or hierarchy of this variable through the application of the alpha weight (α), an extra hour decrease from 315 to 75 was evidenced, that is, a drop of 76.19%. For its part, it also meant a 26.66% increase in hours non-worked, increasing from 900 to 1140 h. These results were in line with what was expected since not giving relative importance to extra hours or non-worked hours, it implied the non-existence of hourly modifications and remained stable at 315 and 900 h, respectively. Fig. 2 presents the behavior of the alpha weight as a function of deviation hours for extra and non-worked but paid hours.

In contrast, for the second scenario where the project gives more importance to the reduction of the non-worked but paid hours, represented by the beta weight (β), results showed an analogous behavior to that described for the alpha weight (α). Because of the two scenarios described, it is possible to establish that the individualized analysis of extra hours and time non-worked yielded valuable results regarding the convenience of prioritizing the minimization of the hourly deviations described, although subject to the reality of each company and/or project. In this regard, for certain construction projects, the need to reduce costs associated with extra hours for employees on-site may be more relevant. However, unproductive work, also called non-contributory work, represents an acute industrial pain for the construction sector [65,66,67]. This is because, unlike extra hours, it is detrimental to the continuity and execution of the stages that make up the project planning. In addition, it corresponds to an implicit cost for the company, which may lead to delays and daily fines, in addition to assuming operational costs for the availability of cranes, water, and energy, besides the required workforce.

#### 4.1.2 Results from experiment Nº2

The evaluation of the work scheduling relevance, compared to the production requirements established by the construction project decision-maker was proposed, to avoid potentially additional costs. For the building project, taking as a baseline condition a correctly developed labor planning, the model contemplated 315 and 900 extra hours and hours non-worked, respectively. Based on the above, the result obtained from the factor gamma (γ) on $\tau_{kj}$ parameter was disaggregated into two instances.

**Fig. 3:**
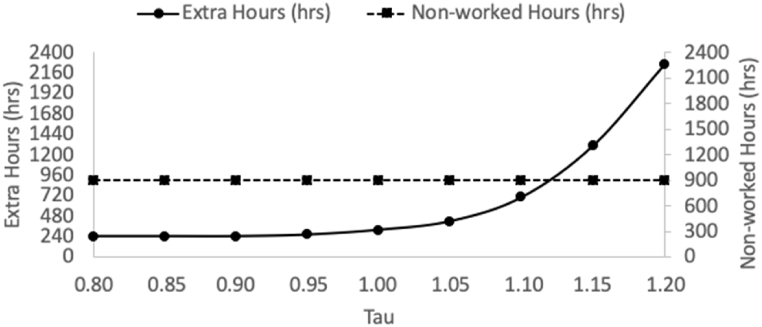
Chart with the analysis of the factor gamma (γ) on $\tau_{kj}$ parameter and its impact on the over or under estimations of labor hours required by the crew.

On the one hand, data obtained over the baseline condition have been interpreted as an overestimation of working hours, in contrast to what is required in the project. In this regard, results revealed an exponential increment in extra hours, reaching 2,250 h from the baseline scenario. In other words, hourly productivity 20% higher than that required by the decision-maker could mean an increase of at least 600% in extra hours. At a budget level, this implies high additional costs, which are avoidable in the face of a properly planned work model. On the other hand, data obtained below the baseline are due to an instance of underestimation of required hours. In contrast to the previous scenario, it was possible to appreciate the existence of 900 non-worked hours but paid, whose behavior is constant even during the overestimation of working hours. This finding is especially relevant since it indicated the existence of a permanent cost associated with non-worked remuneration which the work must assume, and which derives from the labor constraints imposed by the COVID-19 contingency to guarantee the execution of the construction project. Fig. 3 presents the behavior of the factor gamma (γ) on $\tau_{kj}$ parameter, as a function of deviation hours for extra and non-worked but paid hours.

According to what was stated by Serpell et al. [66], the oversizing of labor resources is a critical factor for a construction project. In this regard, there is a general agreement that establishes that construction projects consider more labor than is required per work, for which unqualified workers are usually used. In practical terms, foremen prefer to have a surplus of workers to quickly solve any potential risks that may arise during the on-site work [65,68], which can give rise to asymmetric on-site workforce costs.

#### 4.1.3 Results from experiment Nº3

The ε−constraint method was used to generate a Pareto Frontier, to favor the efficiency relationship analysis concerning the working times of the model. The results showed that, as hours non-worked per employee and week decrease, the extra hours on the construction site increase, being the extreme points where the maximum non-worked time without extra hours is reached, and analogous to the maximum of productive time. The rest of the points on the line correspond to efficient times for the decision-maker, that is, pairs of solutions that deliver the least deviation hours, associating lower work costs in terms of labor scheduling, and that would benefit the construction project of the company (Table 1).

**Table 1:** Set of efficient and non-dominated solutions.

Total extra work hours ($F_{li}^{+}$)	Total non-worked hours ($F_{li}^{-}$)
75	1,140
80	1,135
85	1,130
90	1,125
95	1,120
100	1,115

When proposing the ε-constraint, the sum of extra hours ($F_{li}^{+}$) was used as a new constraint given that, according to the existing literature on lean construction, the non-contributory hours or paid hours for non-worked, correspond to a more relevant variable than productive hours, since they constitute around 53% of the total time spent during a construction site [66].

#### 4.1.4 Results from experiment Nº4

The result of the numerical analysis carried out from the Gantt Chart diagrams with and without the COVID-19 condition made it possible to verify the differences between extra and non-worked but paid hours, and the total time deviation between the scenarios mentioned above. The relevance of this analysis lies in the fact that time differences directly impact new costs and the completion time of the project under construction. Specifically, the elimination of constraints derived from COVID-19 removes the presence of any time deviation, whether productive or unproductive. In practice, this could be interpreted as the correct constructor work execution with an absolute level of productivity, in other words without additional payment (Table 2). However, these results reflect the proposed model, so future work could show possible time deviations even without considering the COVID cost since they consider real data studied from building projects.

**Table 2:** Comparison of hourly deviations associated with workers in the construction project, with and without COVID constraints.

Criteria	With COVID constraint	Without COVID constraint
Total extra work hours ($F_{li}^{+}$)	97	0
Total non-worked hours ($F_{li}^{-}$)	1,118	0

On the other hand, a comparative analysis of the spent working hours, i.e., based on the variable ($y_{kl}^{ij}$), leads to conclude that eliminating COVID-19 constraints favors an increment in working hours of between 1.35 and 4.00%. In line with the previously mentioned, elimination of productive work deviations would theoretically promote an increment in the time invested in work given the greater freedom of movement of employees, along with greater availability to work between weeks, as shown in Table 3.

**Table 3:** Comparison of working hours spent by workers per area in the construction project, with and without COVID constraints.

Area	Total working hours spent per area ($y_{kl}^{ij}$)		Increment
	With COVID constraint	Without COVID constraint	
1	14,504	14,700	1.35%
2	11,250	11,700	4.00%
3	7,500	7,800	4.00%
4	4,725	4,800	1.58%

As mentioned above, construction activities must necessarily be carried out in person and undertaken physically demanding tasks in an outdoor environment [34], given the impossibility of developing remote processes [9]. Faced with this reality, the sector has taken strict safety and sanitation measures to minimize the contagion risk inside works. As a result of the above, projects have shown a considerable decrease in the employees' number duly authorized to work on the construction site, which explains the project's slowdown in contrast to the committed completion times [9].

### 4.2 Model application to the construction industry results

A set of experiments was designed to evaluate the model consistency, based on theoretical data inspired by construction projects, with a flexible working time schema due to COVID circumstances. Specifically, the input data used in this research was obtained from a real residential project consisting of 280 houses, located in an important city in southern Chile. In this case study, the health context generated by the Covid-19 pandemic demanded the implementation of a flexible work system based on a workforce of a 104-man crew, divided into seven specialties. The project's Gantt chart is bidding on a 40-week on-site project, working a maximum of 45 weekly hours per employee, i.e., five days and nine contractual hours each day. In addition, a maximum of 10 weekly extra hours per employee, i.e., 2 h per day, can be completed to summarize up to 55 weekly hours per employee, according to local labor codes. Because of the company's internal policy, the construction project considers only a day shift, from Monday to Friday. It must be mentioned that the model explained in sub-section (3.1) considered a European context, which has more flexible working conditions in terms of weekly hours per employee compared to the case study. Determining an efficiency balance between the workforce scheduling, deviation hours, and potential additional costs were evaluated for two operational scenarios and compared with the original project condition. In Scenario 1, the workforce amount baseline was contrasted in terms of additional employees potentially needed when extra hours are not allowed to exceed the contractual 45 weekly hours per employee. In Scenario 2, an additional amount of employees was contrasted by allowing up to 10 weekly extra hours (Table 4).

**Table 4:** Basic data of the construction project case study for scenarios 1 and 2.

Set	Elements
Weeks	40
Shifts	Monday to Friday morning, Monday to Friday afternoon, and Saturday morning
Man-crew workers baseline	104
Man-crew workers Scenario 1	212
Man-crew workers Scenario 2	182
Specialties	7
Maximum weekly working hours per employee	45
Maximum weekly extra hours per employee	10

When comparing total deviation hours, a sum of extra ($F_{li}^{+}$) and non-worked hours ($F_{li}^{-}$), with the first and second operational scenarios and the crew baseline, results from the proposed model demonstrate that hiring a maximum number of employees can allow the company to avoid extra hours payment. Nevertheless, it gives rise to a significant increment in the non-worked paid hours, as exhibited in Table 5.

**Table 5:** Deviation, extra work, and non-worked hours comparison between two operational scenarios and the construction project baseline, with the COVID constraint.

Type of crew	Number of workers	Extra work hours ($F_{li}^{+}$)	Non-worked hours ($F_{li}^{-}$)
Baseline	104-man	85,860	93,600
Scenario 1	212-man	0	190,800
Scenario 2	182-man	21,780	163,800

The results show that the greatest amount of non-worked or non-contributory work is associated with Scenario 1, being 103.8% and 75.0% greater than the baseline and Scenario 2, respectively. It is necessary to mention that the 212-crew scenario is a theoretical condition where extra hours are not allowed. Nevertheless, unproductive hours are still present as shown by the model. At a qualitative level, it represents not just a risk for accomplishing daily activities [66], but also a potential factor to increase the contagion risk, as a result of employees not doing their work or not being in their work area. On the other hand, the 182-crew scenario associates a low increase in non-worked hours. But, considering the company's need for executing the construction project under the COVID constraint, hiring the lowest number of employees may contribute in terms of having a suitable crew rather than a surplus, to solve operational problems more efficiently as indicated by Lean Construction [65,68]. For both cases, Scenario 1 and 2, by lifting the COVID constraint established by the model, 212-crew and 182-crew would increase the freedom of displacement around the construction site, thus deducing a greater on-site contagion probability because of a greater number of potential viral vectors.

The company's decision-maker needs to determine the COVID cost, derived from hiring new employees who require PPEs, sanitization/hygiene conditions, and/or paying extra hours in line with the Labor Code. Table 6 shows the worker's monthly income.

**Table 6:** Worker's monthly incomes according to the local context (case study).

Specialty	Total number	Income (USD/month)
Laborer	50	27,825.87
Carpenter	16	12,861.74
Welder	2	1,607.72
Electric	12	9,646.30
Builder	8	6,430.87
Ironworker	12	9,646.30
Plumber	4	3,215.43
Total	104	71,234.23

For both scenarios under the COVID constraint, the company must practice social distancing through employees working on specific weeks, along with a sanitization and hygiene policy enabled by incorporating PPEs for employees during the whole 40-week construction project (Table 7).

**Table 7:** COVID-19 PPE monthly cost for a 40-week on-site project.

Equipment	Unit Cost (USD/worker)
Fit test	45
N95 respirator	45
Facial mask	25
Latrines	150
Hand Wash stations	150
Sanitization	30
Total	445

#### 4.2.1 Results from experiment I

In Scenario 1, the 104-man crew basis associates a sum of 90 extra hours per employee by week. This means that, by incorporating the COVID weekly operational constraint for employees during the construction project on-site execution, the company would have to pay up to 7 daily extra hours per employee, which is not possible because it represents a violation of the local labor codes. However, results obtained by applying the model show that a 212-man crew would be required to match the maximum working hours with extra hours per employee by week, i.e., 45 weekly hours. In practice, the decision-maker could save the extra hours payment when adding 108 workers to the original 104 on-site crew (Table 8).

**Table 8:** Extra hours variation when adding new workers and without extra hours allowed.

Specialty	104-man crew		212-man crew	
	Workers number	Weekly hours	Workers number	Weekly hours
Laborer	50	90	100	45
Carpenter	16	90	33	45
Welder	2	90	5	45
Electric	12	90	25	45
Builder	8	90	16	45
Ironworker	12	90	24	45
Plumber	4	90	9	45
Total	104		212	

In Scenario 2, a balance between hiring workers and allowing extra hours is accepted by the company under the proposed COVID constraint. In this regard, results from the mathematical model show that a 182-man crew would be required in the construction project to reach up to 55 weekly hours, in other words, 45 weekly contractual hours plus 10 weekly extra hours per employee (Table 9).

**Table 9:** Extra hours variation when adding new workers and with extra hours allowed.

Specialty	104-man crew		182-man crew	
	Workers number	Weekly hours	Workers number	Weekly hours
Laborer	50	90	85	54
Carpenter	16	90	28	52
Welder	2	90	4	45
Electric	12	90	21	54
Builder	8	90	15	52
Ironworker	12	90	21	54
Plumber	4	90	8	45
Total	104		182	

In detail, hiring 35 laborers makes it possible to reach up to 54 weekly hours, which implies a 7 extra hours payment per laborer. Then, based on the 6,2 USD/hour which is the extra hour value of the local construction market of the case study, the decision maker could save up to 223 USD/month. Similar occurs for carpenters, electrics, ironworkers, and builders. Particularly for plumbers, results from the model indicate that there is no need to perform extra work if four additional employees are added. The same happens to welders, where hiring two additional employees leads the company to not require extra hours. The latter is explained by the match between the maximum weekly hours with the weekly extra hours. In practice, adding 78 new workers to the original 104 on-site crew would lead the decision-maker to pay up to 1.014 USD/month just for extra hours (Table 10).

**Table 10:** Extra hours variation when adding new workers and with extra hours allowed.

Specialty	Extra hours (hours/week)	Extra hours payment (USD/week)	Extra hours payment (USD/mth)
Laborer	9	55.65	222.61
Carpenter	7	43.28	173.14
Welder	0	0	0
Electric	9	55.65	222.61
Builder	7	43.28	173.14
Ironworker	9	55.65	222.61
Plumber	0	0	0
Total		253.52	1,014.10

#### 4.2.2 Results from experiment II

For Scenario 1, adding 108 new workers over the 104-man crew avoids extra hours payment, but leads to an increase in the monthly income and PPE, due to the sanitization and hygiene COVID policy adopted by the company (Table 11).

**Table 11:** Additional costs derived from hiring workers to set up a 212-man crew under the COVID constraint.

Specialty	Income (USD/mth)	PPE (USD/mth)	Total cost (USD/mth)
Laborer	27,825.87	22,250.00	50,075.87
Carpenter	13,665.59	7,565.00	21,230.59
Welder	2,411.58	1,335.00	3,746.58
Electric	10,450.16	5,785.00	16,235.16
Builder	6,430.87	3,560.00	9,990.87
Ironworker	9,646.30	5,340.00	14,986.30
Plumber	4,019.29	2,225.00	6,244.29
Total	74,449.67	48,060.00	122,509.67

It is necessary to highlight that Table 5 corresponds to the monthly income for a 104-man crew baseline without considering the COVID pandemic, while Table 10 presents additional costs as a result of hiring additional workers because of implementing COVID restrictions and therefore requiring monthly PPEs per worker. For specialties such as laborer, builder, and ironworker, there are no additional costs related to hiring workers nor PPE required for new employees. On the contrary, there are hiring and PPEs extra costs associated with specialties like carpenter, welder, electric, and plumber, up to reaching a 212-man crew, according to the results given by the mathematical model. This income difference is because there are specialties more sensible to the COVID restrictions applied for the case study, where extra workers are needed.

For Scenario 2, adding 78 new employees over the 104-man crew also leads to an increase in the monthly income and PPE. However, extra hours payment must be made by the company according to the local labor codes (Table 12).

**Table 12:** Additional costs derived from hiring workers to set up a 182-man crew, and working extra hours under the COVID constraint.

Specialty	Salary Income (USD/mth)	PPE (USD/mth)	Extra hours income (USD/mth)	Total cost (USD/mth)
Laborer	19,478.11	15,575.00	222.61	35,275.72
Carpenter	9,646.30	5,340.00	173.14	15,159.44
Welder	1,607.72	890.00	–	2,497.72
Electric	7,234.73	4,005.00	222.61	11,462.33
Builder	5,627.01	3,115.00	173.14	8,915.15
Ironworker	7,234.73	4,005.00	222.61	11,462.33
Plumber	3,215.43	1,780.00	–	4,995.43
Total	54,044.03	34,710.00	1,014.10	89,768.13

According to the needs of the decision-maker regarding quantifying the COVID cost, a total cost comparison shows that the COVID constraint leads the company to increase both income and sanitization/hygiene costs for the 40-week project. Nevertheless, the 182-man crew provided by Scenario 2 represents a 76.39% additional cost in contrast to a 104.25% increment associated with the 212-man crew for Scenario 1 (Table 13).

**Table 13:** Total cost comparison between Scenario 1, Scenario 2, and the baseline crew of the original construction project under the COVID constraint.

Specialty	Total Cost (USD)		
	104-man crew	212-man crew	182-man crew
Laborer	50,075.87	100,151.74	85,351.59
Carpenter	19,981.74	41,212.33	35,141.18
Welder	2,497.72	6,244.29	4,995.43
Electric	14,986.30	31,221.46	26,448.64
Builder	9,990.87	19,981.74	18,906.02
Ironworker	14,986.30	29,972.60	26,448.64
Plumber	4,995.43	11,239.73	9,990.87
Total	117,514.23	240,023.90	207,282.36
% Inc		104.25	76.39

### 4.3 Discussion and implications for practitioners

The model application to a real study case has demonstrated to be a reliable tool for identifying COVID costs derived from executing a project during a pandemic event. Even when different results were obtained through different operational strategies, like hiring employees to avoid extra hours payment, or prioritizing small amounts of additional people to reduce sanitization or incomes costs; new research and publications concerning companies delimiting crews per area, should be made toward measuring the COVID-19 effects and costs on the construction sector.

In addition, it is important to emphasize that advanced optimization algorithms are becoming increasingly important in solving challenging decision-making problems that involve large data sets, complex constraints, or multiple objectives. On the other hand, traditional optimization algorithms may not be able to efficiently handle such problems, which is where advanced optimization algorithms come into play. Evolutionary metaheuristics, memetic algorithms, island algorithms, and multi-objective specialized metaheuristics are some of the most promising advanced optimization algorithms that can solve large-scale and complex decision-making problems.

In this sense, it is also possible to consider the evolutionary metaheuristic algorithms [69], which use natural selection and genetics principles to effectively search through large solution spaces and find optimal or near-optimal solutions for complex decision-making problems. In addition, memetic algorithms [70,71] combine global and local search methods to find suitable solutions to optimization problems, making them well-suited for problems with complex constraints. Alternatively, island algorithms [72] which consider the partition of a large search space into smaller subspaces, allowing for parallel processing of smaller subproblems, are useful when looking for a faster convergence to a good solution.

Despite the above-mentioned optimization methods, the multi-objective specialized metaheuristic algorithms [73,74], designed specifically for problems with multiple objectives, can also be very effective in solving decision-making problems with conflicting objectives. Thus, the multi-objective optimization mathematical model used in the present research is an effective way to assess the construction project's efficiency, taking into account the employee workforce scheduling and the associated COVID costs, during the on-site work completion under a pandemic context.

From a theoretical and methodological perspective, this research may contribute to the body of knowledge by providing a practical tool to assess the workforce scheduling efficiency in construction projects by using a multi-objective optimization model under a more complex scenario triggered by a pandemic context. In terms of implications for practice, this study aims to provide an optimization tool that may help project and construction managers to prioritize the number of extra hours or non-worked hours according to the available budget in a project, avoiding labor overestimations that may increase the extra hours paid.

### 4.4 Limitations and future research

Despite the previous findings, in terms of limitations and future research, it has to be noted that considering a wider range of shift patterns and work arrangements, such as night shifts, split shifts, or irregular shifts —as part of the paradigms that the construction industry is facing today triggered by new challenges (pandemics, climate change, and others)—, could be an important way for future research in construction scheduling. In this sense, developing scheduling models that are more flexible and adaptable to different types of shifts could also help optimize scheduling decisions more realistically and practically, considering the multiple work schedules often found in the construction industry.

Additionally, incorporating more comprehensive productivity measures into the scheduling model could enhance its accuracy and effectiveness. Considering factors such as skill levels, task difficulty, or other relevant productivity indicators could provide a more holistic understanding of productivity in the construction industry. On the other hand, developing models that can capture this holistic view —as a result of a multi-objective optimization approach—, and integrate it into the scheduling process could drive more accurate and informed scheduling decisions, leading to better resource allocation and project performance.

Overall, addressing the limitations related to shift flexibility and productivity measures through further research and model development could contribute to the advancement of construction scheduling techniques, making them more adaptable and reflective of the complex realities of the construction industry.

## 5 Conclusion

During the development of this research, a multi-objective optimization mathematical model was proposed to evaluate employees’ hourly working schedules in the construction sector, during the COVID-19 pandemic context.

Results from the design of the experiments conducted show that prioritization of extra hours or non-worked but equally paid hours is convenient according to the budget and operational reality of each company and/or project. Regarding the worker's scheduling importance, it is established that labor overestimation induces an exponential increase in extra hours and costs, contrary to the linear trend of unproductive hours. In addition, there is a permanent number of non-worked hours, as a result of maintaining the work under the new constraint. For its part, it is shown that the decrease in non-worked hours is exchanged for the increase in extra hours. The numerical analysis verifies the presence of a COVID-19 cost by eliminating extra and non-worked hours in general. Similarly, a productive labor increase can be seen when COVID constraints are lifted since it leads to an increase in time on site given greater weekly availability.

As a consequence, the findings confirm that unproductive work or non-contributory work —in terms of non-worked hours—, represents one of the most challenging issues that the construction industry has to deal with. Thus, the multi-objective optimization model proposed in this research may help avoid having more labor than is required per work and eliminate the controversial belief that it is always beneficial to have more manpower in case something goes wrong.

The proposed mathematical model represents a theoretical and practical tool for decision-making support, regarding the effects and costs of COVID-19 on labor scheduling in works within the construction sector.

Finally, future work should focus on using machine learning to identify patterns in data related to COVID-19 constraints and their impact on the construction industry. This could allow for more adaptive and responsive decision-making in a dynamic environment. Additionally, it would be valuable to investigate the transferability of the proposed methodology to different quarantine policies and labor regulations. Furthermore, exploring the use of advanced optimization algorithms, such as adaptive heuristics and metaheuristics, could enable more efficient and effective workforce scheduling for larger-scale instances.

## Declaration of competing interest

The authors declare that they have no known competing financial interests or personal relationships that could have appeared to influence the work reported in this paper.
